# Clinical Efficacy and Safety of Buyang Huanwu Decoction for Acute Ischemic Stroke: A Systematic Review and Meta-Analysis of 19 Randomized Controlled Trials

**DOI:** 10.1155/2012/630124

**Published:** 2012-10-21

**Authors:** Chi-zi Hao, Fan Wu, Jiangang Shen, Lin Lu, Deng-lei Fu, Wei-jing Liao, Guo-qing Zheng

**Affiliations:** ^1^Department of Rehabilitation, Zhongnan Hospital of Wuhan University, Wuhan 430071, China; ^2^The Center of Neurology and Rehabilitation, The Second Affiliated Hospital of Wenzhou Medical College, Wenzhou 325027, China; ^3^School of Chinese Medicine, University of Hong Kong, Hong Kong

## Abstract

Buyang Huanwu Decoction (BHD) is a well-known traditional Chinese herbal prescription for treating stroke-induced disability. The objective of this study was to evaluate the efficacy and safety of BHD for acute ischemic stroke. A systematic literature search was performed in 6 databases until February 2012. Randomized controlled clinical trials (RCTs) that evaluate efficacy and safety of BHD for acute ischemic stroke were included. Nineteen RCTs with 1580 individuals were identified. The studies were generally of low methodological quality. Only one of the trial included death or dependency as a primary outcome measure. Only 4 trials reported adverse events. Meta-analysis showed the clinical effective rate of neurological deficit improvement favoring BHD when compared with western conventional medicines (WCM), *P* < 0.001. There is significant difference in the neurologic deficit score between the BHD treatment group and the WCM control group, *P* < 0.001. In Conclusion, BHD appears to improve neurological deficit and seems generally safe in patients with acute ischemic stroke. However, the current evidence is insufficient to support a routine use of BHD for acute ischemic stroke due to the poor methodological quality and lack of adequate safety data of the included studies. Further rigorously designed trials are required.

## 1. Introduction

Stroke is one of the major causes of disability and dependence in the world [1], and WHO estimated that it accounts for 5.7 million deaths worldwide in 2005, which is equivalent to 9.9% of all deaths [2]. The rates of stroke mortality and burden vary greatly among countries, but low-income countries are the most affected [3]. Ischemic stroke was the most common subtype, accounting for about 80% of all strokes. However, the optimization of modern clinical treatment with acute ischemic stroke was only an integrated and systematic approach with thrombolysis, if indicated, and aggressive supportive care [4]. Therefore, the rising number of stroke patients resorts to various kinds of complementary and/or alternative medicine (CAM) worldwide.

 China, as a developing country, has the largest number of stroke cases in the world because it has a population of 1.34 billion in 2011. The most appreciable difference between China and the Western countries in treating stroke is the use of Traditional Chinese Medicine (TCM) therapy including Chinese herbal medicine (CHM), acupuncture, and other nonmedication therapies [5]. Buyang Huanwu Decoction(BHD) is a well-known classic TCM herbal prescription for ischemic stroke and has been used for functional recovery of stroke-induced disability for more than 200 years [6]. BHD is composed of seven kinds of Chinese medicine: Huangqi (Radix Astragali seu Hedysari), Danggui (Radix Angelicae Sinensis), Chishao (Radix Paeoniae Rubra), Chuanxiong (Rhizoma Ligustici Chuanxiong), Honghua (Flos Carthami), Taoren (Semen Persicae), and Dilong (Pheretima), all of which are recorded in the Chinese Pharmacopoeia. In modern time, BHD is still widely used throughout China and elsewhere in the world for the treatment of ischemic stroke. Experimental studies indicate that BHD has neuroprotective and neurogenesis-promoting effects. Main findings are as the follows: protecting neurons from ischemic injury [7, 8], promoting the regeneration of peripheral nerves [9] and differentiation of neural progenitor cells [10], improving recovery of neurological function, reducing infarction volume, stimulating neural proliferation [11], and repairing the injured blood vessels and lesion tissues [12]. However, there is still a lack of reliable scientific evidence for BHD treatment in patients with ischemic stroke. 

 BHD is commonly used in the acute, recovery, and sequelae stages of patients with ischemic stroke in China. However, the most important period of recovery is at the acute and subacute stages during the clinical course of ischemic stroke [13]. In this study, we aimed to evaluate the clinical efficacy and safety of BHD therapy for patients suffering from acute ischemic stroke within the first seven days of onset.

## 2. Methods

### 2.1. Eligibility Criteria

**Types of Studies**

Randomized controlled clinical trials (RCTs) that evaluate efficacy and safety of BHD for ischemic stroke patients were included. Quasi-RCTs were not considered such as using the admission sequence for treatment allocation.

**Types of Participants**

Patients of any gender, age, or race/ethnicity with ischemic stroke within 7 days of onset were considered. The ischemic stroke was diagnosed clinically according to the World Health Organization definition [14] or the diagnostic criteria issued at the Second and revised at the Fourth National Cerebrovascular Diseases Conference in China [15, 16] and approved by CT scan or MRI.

**Types of Interventions**

The patients of the control group were given western conventional medicines (WCM). WCM refer to the combination of needed therapies of the following aspects: (1) general supportive care mainly includes (A) airway, ventilatory support and supplemental oxygen, (B) cardiac monitoring and treatment, (C) temperature, (D) blood pressure, (E) blood sugar, and (F) nutrition; (2) specialized care mainly includes a variety of measures to improve cerebral blood circulation (such as antiplatelet agents, anticoagulants, fibrinogen-depleting agents, volume expansion, and vasodilators, except thrombolytic agents) and neuroprotective agents; (3) treatment of acute complications mainly includes (A) brain edema and elevated intracranial pressure, (B) seizures, (C) dysphagia, (D) pneumonia, (E) voiding dysfunction and urinary tract infections, and (F) deep vein thrombosis. The intervention for control group included only WCM treatments. Studies comparing BHD therapy to another form of Chinese herbal medicine were excluded. The patients at the treatment groups were given BHD therapy in addition to WCM which was similar to the control group. Modified BHD was (BHD plus few herbal) also included. The clinical trials were included regardless of length of treatment period and dosage of treatment.

**Types of Outcome Measures**

The primary outcome measures were death or dependency at the end of followup (at least 3 months). Dependency was defined as need assistance in activity of daily living, such as the Barthel Index ≤60. The secondary outcome measures were the neurological deficit improvement after treatment and adverse events. The scores of neurological deficit improvement and the effective rate were both considered in the neurological deficit improvement.

In this paper, the neurologic deficit score criteria were adopted based on the Modified Edinburgh-Scandinavian Stroke Scale, a nationwide accepted scoring system recommended at the Second and revised at the Fourth National Cerebrovascular Diseases Conference in China [17], including consciousness, gaze, facial paresis, language, walking ability, motor function of arms, legs, and hands. The effective rate was conducted in accordance with the Modified Edinburgh-Scandinavian Stroke Scale, which classified disability into five categories as cure (the scores of functional deficit were decreased up to 91–100%, and disability degree was at grade 0), significant improvement (the scores of functional deficit were decreased at 46–90%, and disability degree was at the grade 1–3), improvement (the scores of functional deficit were decreased at 18–45%), no improvement (the scores of functional deficit were decreased at about 17%), and deterioration (the scores of functional deficit were increased over 18%). Moreover, it was dichotomized as effective (including the categories of cure, significant improvement, and improvement) and ineffective (including the categories of no improvement and deterioration) [17].

### 2.2. Information Sources and Search

We searched Cochrane library; PubMed; EMBASE; China National Knowledge Infrastructure; VIP Journals Database; Wanfang database until February 2012. The search terms used were (Bu-yang Huan-wu decoction OR Bu-yang-Huan-wu decoction) AND (Ischemic Stroke OR Cerebral infarction OR cerebral embolism); Chinese Databases were also searched using the above search terms in Chinese. We hand-searched Chinese journals that may publish potentially eligible studies and conference proceedings relevant to this topic. The reference lists of all relevant articles were searched for further studies.

### 2.3. Study Selection and Data Collection Process

All articles were read by two independent reviewers (Hao CZ, Wu F), who extracted data from the articles according to a standardized data extraction form, including patients, methods, interventions, and outcomes. The reasons for the exclusion of studies were recorded accordingly. For eligible studies, two review authors (Hao CZ, Wu F) extracted the data independently. Disagreements were resolved through consultation with a third party author (Zheng GQ or Liao WJ).

### 2.4. Risk of Bias in Individual Studies

The risk of bias was assessed using the twelve criteria recommended by the Cochrane Back Review Group [18], and the level of evidence was assessed by the GRADE system [19–22]. Disagreements were resolved by discussion between the two reviewers (C.-z. Hao, F. Wu), with the opinion of a third party author (G.-q. Zheng or W.-j. Liao) if necessary.

### 2.5. Summary Measures and Synthesis of Results

We synthesized the results in a meta-analysis. A fixed-effects model or random-effect model was used across the trials, and risk ratios with their 95% confidence intervals (CI) were calculated for dichotomous data. If continuous data were available, weighted mean difference or standardized mean difference was to be calculated using RevMan 5.1 software provided by the Cochrane Collaboration, and Cochrane's *Q*-test. I2 were used to assess heterogeneity. Where possible, we assessed publication bias using a funnel plot.

## 3. Results

### 3.1. Study Selection

**Figure 1:**
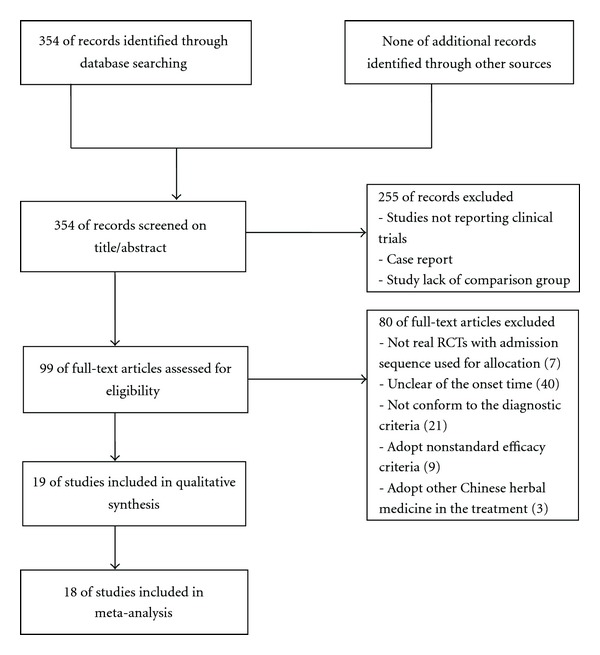
Flowchart of trials selection process.

On the basis of search strategy, we identified 354 potentially relevant articles, and 255 articles were excluded because they were not reporting clinical trials, case report, or lacking comparison group. Of the remaining 99 articles, 80 were excluded because 7 articles were not real RCTs with admission sequence used for treatment allocation, 61 with patients who did not meet the criteria of the types of participants; there are 3 trials used Chinese Herbal Injections in control group and 9 adopted nonstandard efficacy criteria. Finally, 19 studies, involving a total of 1580 participants, met our inclusion criteria [23–41]. The screening process is summarized in a flow diagram (Figure 1). 

### 3.2. Study Characteristics

The 19 studies included were all conducted in China and published between 1995 and 2012, and all of them were performed in a single center. The sample size was small, with 3 having a size less than 50, and 11 between 50 and 100, the other 5 between 100 and 200, and none reported sample size estimation. All the 19 RCTs based the diagnosis of acute ischemic stroke on both clinical examination and CT or MRI. All the 19 RCTs used BHD combining with conventional western therapy as the treatment group, and conventional western therapy as control group. The duration of studies lasted from 10 days to 30 days. Both clinical effective rate and neurologic deficit scores were observed in 8 studies, while only clinical effective rate was observed in 9 studies, only neurologic deficit scores were observed in 1 studies [28], and one study observed the ESS neurologic deficit scores [29]. Adverse effects were reported in 4 studies [29, 32, 38, 39], while the other 15 included trials not mentioning adverse events at all. Key data are summarized in Table 1.

### 3.3. Risk of Bias within Studies

All of the studies were described as randomized, but no study reported the method of random sequences generation. No study mention allocation concealment. Only one study mentioned single blinding [39], but not did mentioned either subjects or investigator or assessor blinding. None of the studies described intention-to-treat analyses, and no study reported follow-up or dropout data. In general, all 19 RCTs showed an unclear risk of bias based on the Cochrane Risk of Bias tool (Table 2).

Based on GRADE system, the evidence of effective rate and neurological deficit scores (Modified Edinburgh-Scandinavian Stroke Scale) was level D, while the evidences of neurological deficit scores (ESS) was level C, and all of them were weak recommendation (Table 3).

### 3.4. Results of Individual Studies

#### 3.4.1. Death or Dependency

One study used the Barthel Index to evaluate the dependency rate [31]. The result indicated that there was no statistically significant difference between the two groups (Peto OR, 0.36; 95% CI, 0.12 to 1.07). Only one trial reported one case of death in the control group. None of the studies reported the mortality and dependency at the end of followup (at least three months).

#### 3.4.2. Adverse Events

4 studies reported that there were no adverse events [29, 32, 38, 39], while the left 15 studies did not mention adverse events. However, no life threatening adverse effects were noted in these studies.

### 3.5. Synthesis of Results

#### 3.5.1. The Effective Rate of Neurological Deficit Improvement

**Figure 2:**
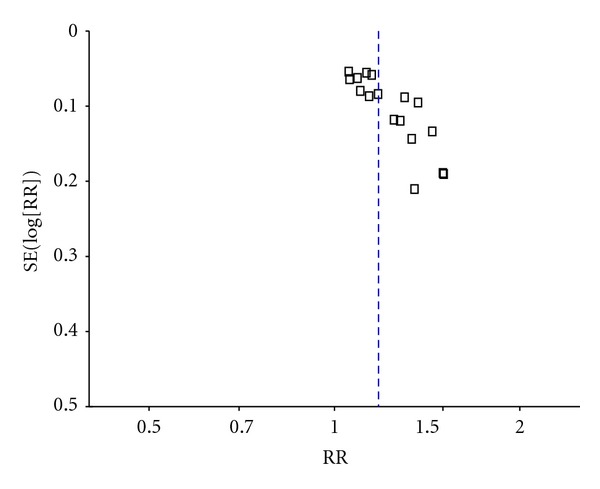
Funnel plot of the total effective rate of BHD therapy for acute ischemic stroke.

17 of the included studies which adopted the effective rate to assess the clinical improvement were qualified to perform a meta-analysis, and the random-effect model was used for statistical analysis because of the heterogeneity (*n* = 1444, RR 1.18, 95% CI 1.12 to 1.24, *P* < 0.001, heterogeneity *χ* ^2^ = 24.82, *P* = 0.07, *I* ^2^ = 36%) favoring BHD (Table 4). The publication bias funnel plot provided evidence of publication bias (Figure 2).

#### 3.5.2. The Neurologic Deficit Score

**Figure 3:**
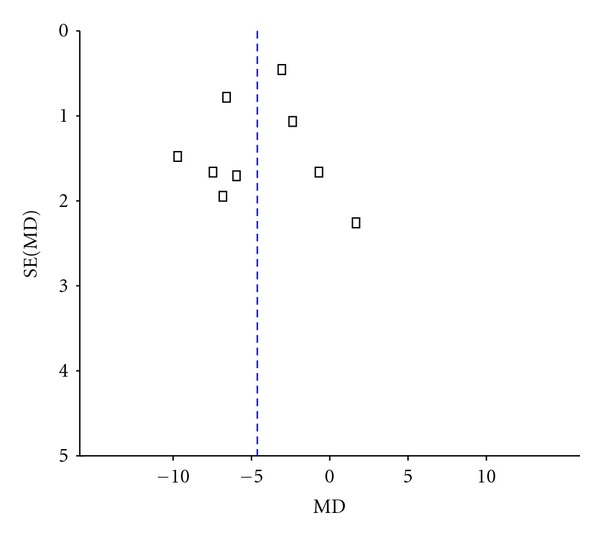
Funnel plot of the scores of neurological deficit of BHD therapy for acute ischemic stroke.

9 studies which used the neurologic deficit score were qualified to perform a meta analysis, and the random-effect model was used for statistical analysis because of the heterogeneity (*n* = 786, MD −4.65, 95% CI −6.57 to −2.72, *P* < 0.001, heterogeneity *χ* ^2^ = 49.62, *P* < 0.001, *I* ^2^ = 84%), and showed the significant difference between the BHD therapy group and the control group and favored BHD (Table 5). We considered high heterogeneity because of *I* ^2^ = 84%. Analysis of the study and patient characteristics suggests the heterogeneity may arise from different course of treatment among the studies (10 days to 30 days). So we divide the 9 studies into 2 subgroups according to the course of treatment (≦15 days, or >15 days). But the results show that heterogeneity does not decrease (*I* ^2^ = 87% and *I* ^2^ = 83%). So we consider that heterogeneity may arise from other substantial heterogeneity. The publication bias funnel plot provided evidence of publication bias (Figure 3). One study [29] adopted the ESS indicated that the improvement of the neurologic deficit scores of BHD group was significantly higher than that of control group (*P* < 0.01). 

## 4. Discussion

### 4.1. Summary of Evidence

Nineteen studies with 1580 individuals suffering from acute ischemic stroke were selected out for this systematic review on the mortality and dependency, clinical efficacy, and safety of BHD treatment for acute ischemic stroke. The main finding of this review was that BHD therapy could improve the neurological deficit of acute ischemic stroke. However, a clinical recommendation cannot be warranted because of the generally low methodological quality of the included studies. Another finding suggested that there was no evidence available about the effect of BHD therapy on the primary outcomes because none of the studies included the rates of death or dependency at the end of followup (at least 3 months). At last, BHD therapy was generally safe for acute ischemic stroke. However, adverse reactions should be rigorously investigated to assess the safety because only 21.1% studies mentioned the safety of BHD therapy.

### 4.2. Limitations

Firstly, there are also a number of methodological limitations in this systematic review. None of the included trials reported the random method or allocation concealment, which may produce selection bias. Only one study mentioned blinding, but did not mention either subjects or investigator or assessor blinding. None of the studies described intention-to-treat analyses, and no study reported follow-up or dropout data; all of which are likely to show exaggerated treatment effects. Moreover, it is well established that 5 to 6 months after stroke are an appropriate time point at which to measure neurological and functional outcome, for spontaneous recovery does not reach a plateau until 5 to 6 months after stroke [42]. But all of the trials evaluated the efficacy immediately after completing the treatment, and the period of followup was not long enough to evaluate the long-term effect of BHD treatment.

Secondly, the testified intervention should be compared with placebo-controlled or current “gold standard treatment” rather than randomly chosen unproved treatment [42]. All studies included in this review used an “A + B versus B” design where patients were randomized to receive a BHD adjuvant therapy plus WCM versus WCM. None of the trials chose any sham or placebo as control, and the WCMs were not “gold standard treatment” but randomly chosen unproved treatment, which raises potential to bias.

Thirdly, the outcome measure of most of the trials was defined as an “effective rate,” the validity and reliability of which were uncertain in assessing the outcome. The primary outcome measure should be focused on the level of activities rather than a vague effective rate [42]. The fatality rate in the primary trials was too low to be reliable, because only 1 trial reported the occurrence of one case death. Although there are several possibilities [43], such as a truly low case fatality rate for ischemic stroke in China, the patients with severe stroke were not sent to hospitals (admission bias), a reluctance of researchers to include severe strokes in research studies (selection bias) or failure to report major outcome events (reporting bias), and only trials with low mortality rates submitted their results for publication (publication bias), but the most probable attribution was lack of true randomization.

Lastly, we made an effort to identify all relevant studies, including those in the West and the East. However, all the studies met the criteria were from China, and this may limit the generalizability of the findings.

## 5. Conclusion

This systematic review provides suggestive evidence for the effectiveness and safety of BHD adjuvant therapy to disability improvement after acute ischemic stroke. However, a clinical recommendation cannot be warranted because of the generally unclear methodological quality of the included studies. We did not find sufficient evidence on the primary measure of efficacy to support the routine use of BHD therapy for ischemic stroke. BHD therapy may have beneficial effects on neurological impairment for ischemic stroke, but this efficacy needs to be further confirmed by methodologically rigorous trials. Therefore, further RCTs with adequate concealment of allocation, double-blinding, placebo-controlled, and long-term followup are needed and should be reported in detail according to the consolidated standards of reporting trials (CONSORT) 2010 statement [44].

## Figures and Tables

**Table 1:** Summary of the characteristics of the included trials.

First author year	Subjects (trial/control)	Age (years)		Intervention		Main outcome measures	Course of disease (d)	Course of treatment (d)
		Trial group	Control group	Trial group+	Control group			
Zhang 2010 [12]	82/82	51–77	50–78	BHD*	WCM**	TER, NDS	<7	30
Wu 2011 [23]	35/35	59.8 ± 7.3	60.5 ± 8.1	BHD* plus two-toothed achyranthes root and syndrome differentiation	WCM**	TER	<1	28
Zhang 2004 [24]	40/40	45–70	42–80	BHD* plus syndrome differentiation	WCM**	TER, NDS	<3	15
Guo 2009 [25]	57/30	48–78	47–80	BHD* plus syndrome differentiation	WCM**	TER, NDS	<3	15
Jia 2010 [26]	32/28	31–70	30–70	BHD*	WCM**	TER	<3	14
Fang 2005 [27]	65/72	68.7 ± 0.8	67.2 ± 0.7	BHD* plus stiff silkworm	WCM**	TER	<3	30
Zhang 2012 [28]	34/36	64.8 ± 5.6	65.2 ± 5.2	BHD* plus pangolin scales, grassleaf sweetflag rhizome, milkwort root, stiff silkworm, two-toothed achyranthes root, and bile arisaema based on syndrome differentiation	WCM**	TER	<2	45
Li 2011 [29]	33/33	75–81	75–80	BHD* plus syndrome differentiation	WCM**	ESS	<3	14
Kang 2006 [30]	36/38	48–80	49–82	BHD* plus danshen root	WCM**	TER, NDS	<3	15
Lin 2008 [31]	32/30	50–75	50–75	BHD* plus scorpion and leech	WCM**	TER	<3	nr
Chen 2007 [32]	33/32	61.6 ± 4.7	58.7 ± 5.6	BHD*	WCM**	TER, NDS	<3	14
Yan 2004 [33]	60/60	46–78	46–80	BHD* plus tangshen	WCM**	TER	<7	20
Cui 2005 [34]	50/30	55–71	56–68	BHD* plus tangshen, fragrant solomonseal rhizome, common aucklandia root, bile arisaema, and white mustard seed based on syndrome differentiation	WCM**	TER	<7	20
Liu 2010 [35]	55/55	39–74	39–75	BHD* plus danshen root and plus syndrome differentiation	WCM**	TER	<3	14
Shi 1995 [36]	21/20	62.9 ± 7.5	63.3 ± 11.9	BHD* plus cassia twig and danshen root	WCM**	TER	<3	10
Run 2001 [37]	24/24	48–76	45–77	BHD* plus syndrome differentiation	WCM**	TER	<3	28
Wang 2005 [38]	64/64	36–65	40–71	BHD* plus stiff silkworm, cicada slough, bile arisaema, grassleaf sweetflag rhizome, and syndrome differentiation	WCM**	TER, NDS	<7	14
Lv 2009 [39]	35/35	64.71 ± 10.63	63.31 ± 10.47	BHD* plus two-toothed achyranthes root and syndrome differentiation	WCM**	TER, NDS	<3	30
Zheng 2004 [40]	27/22	65.6 ± 6.3	62.6 ± 6.8	BHD*	WCM**	TER, NDS	<3	21

Notes: BHD: buyang huanwu decoction, WCM: western conventional medicines, TER: total effective rate, NDS: neurological deficit score; +: mean same as the control group treatment. *BHD is composed of seven kinds of Chinese medicine: Huangqi (Radix Astragali seu Hedysari), Danggui (Radix Angelicae Sinensis), Chishao (Radix Paeoniae Rubra), Chuanxiong (Rhizoma Ligustici Chuanxiong), Honghua (Flos Carthami), Taoren (Semen Persicae), and Dilong (Pheretima). **WCM refer to the combination of needed therapies of the following aspects: (1) general supportive care mainly include: (A) airway, ventilatory support, and supplemental oxygen, (B) cardiac monitoring and treatment, (C) temperature, (D) blood pressure, E. blood sugar, and F. nutrition; (2) specialized care mainly include a variety of measures to improve cerebral blood circulation (such as antiplatelet agents, anticoagulants, fibrinogen-depleting agents, volume expansion, and vasodilators, except thrombolytic agents) and neuroprotective agents; (3) treatment of acute complications mainly include: (A) brain edema and elevated intracranial pressure, (B) seizures, (C) dysphagia, (D) pneumonia, E.voiding dysfunction, and urinary tract infections and F. deep vein thrombosis.

**Table 2:** The methodological quality of the included trials.

First author year	A	B	C	D	E	F	G	H	I	J	K	L
Zhang 2010 [12]	?	?	−	−	?	−	−	?	+	+	+	+
Wu 2011 [23]	?	?	−	−	?	−	−	?	+	+	+	+
Zhang 2004 [24]	?	?	−	−	?	−	−	?	+	+	+	+
Guo 2009 [25]	?	?	−	−	?	−	−	?	+	+	+	+
Jia 2010 [26]	?	?	−	−	?	−	−	?	+	+	+	+
Fang 2005 [27]	?	?	−	−	?	−	−	?	+	+	+	+
Zhang 2012 [28]	?	?	−	−	?	−	−	?	+	+	+	+
Li 2011 [29]	?	?	−	−	?	−	−	?	+	+	+	+
Kang 2006 [30]	?	?	−	−	?	−	−	?	+	+	+	+
Lin 2008 [31]	?	?	−	−	?	−	−	?	+	+	+	+
Chen 2007 [32]	?	?	−	−	?	−	−	?	+	+	+	+
Yan 2004 [33]	?	?	−	−	?	−	−	?	+	+	+	+
Cui 2005 [34]	?	?	−	−	?	−	−	?	+	+	+	+
Liu 2010 [35]	?	?	−	−	?	−	−	?	+	+	+	+
Shi 1995 [36]	?	?	−	−	?	−	−	?	+	+	+	+
Run 2001 [37]	?	?	−	−	?	−	−	?	+	+	+	+
Wang 2005 [38]	?	?	−	−	?	−	−	?	+	+	+	+
Lv 2009 [39]	?	?	?	?	?	−	−	?	+	+	+	+
Zheng 2004 [40]	?	?	−	−	?	−	−	?	+	+	+	+

A: adequate sequence generation; B: concealment of allocation; C: blinding (patient); D: blinding (investigator); E: blinding (assessor); F: incomplete outcome data addressed (ITT analysis); G: incomplete outcome data addressed (dropouts); H: free of selective reporting; I: similarity at baseline; J: cointerventions constant; K: compliance acceptable; L: timing outcome assessments. ^+^Yes, ^−^No, ^?^Unclear.

**Table 3:** Summary of GRADE on evidences of outcomes of Bu-yang Huan-wu decoction for acute ischemic stroke.

Quality assessment							Number of patients		Effect		Quality	Importance
Number of studies	Design	Risk of bias	Inconsistency	Indirectness	Imprecision	Other considerations	Trial	Control	Relative (95% CI)	Absolute		
Effective rate												

17	Randomized trials	Very serious	No serious inconsistency	No serious indirectness	No serious imprecision	Reporting bias	696/747 (93.2%)	535/697 (76.8%)	RR 1.18 (1.12 to 1.24)	138 more per 1000 (from 92 more to 184 more)	*⨁ * **○○○** very low	Important
								75%		135 more per 1000 (from 90 more to 180 more)		

Neurological deficit scores (Modified Edinburgh–Scandinavian Stroke Scale)												

9	Randomized trials	Very serious	No serious inconsistency	No serious indirectness	No serious imprecision	Reporting bias	409	377	—	MD 4.65 lower (6.57 to 2.72 lower)	*⨁ * **○○○** very low	Important

Neurological deficit scores (ESS)												

1	Randomized trials	Very serious	No serious inconsistency	No serious indirectness	No serious imprecision	None	33	33	—	MD 7.99 higher (3.96 to 12.02 higher)	*⨁⨁ * **○○** low	Important

**Table 4:** Meta-analyses of the total effective rate of BHD therapy for acute ischemic stroke.

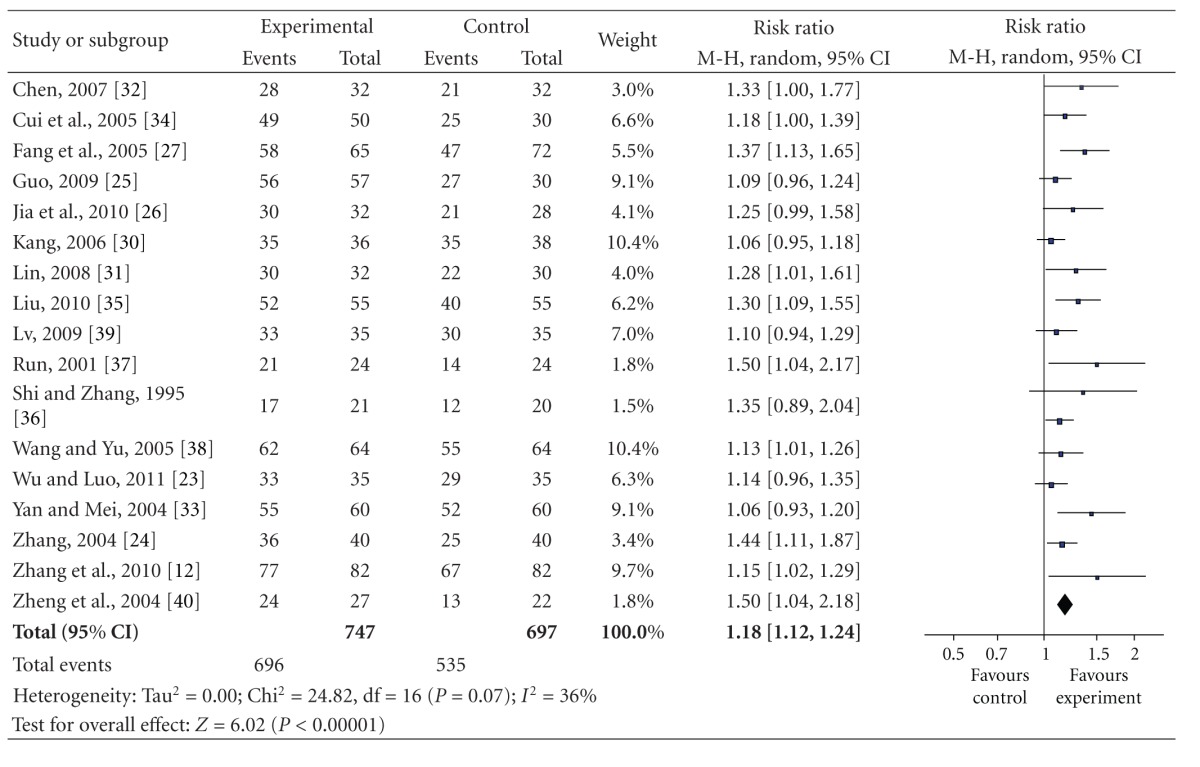

**Table 5:** Meta-analyses of the scores of neurological deficit of BHD therapy for acute ischemic stroke.

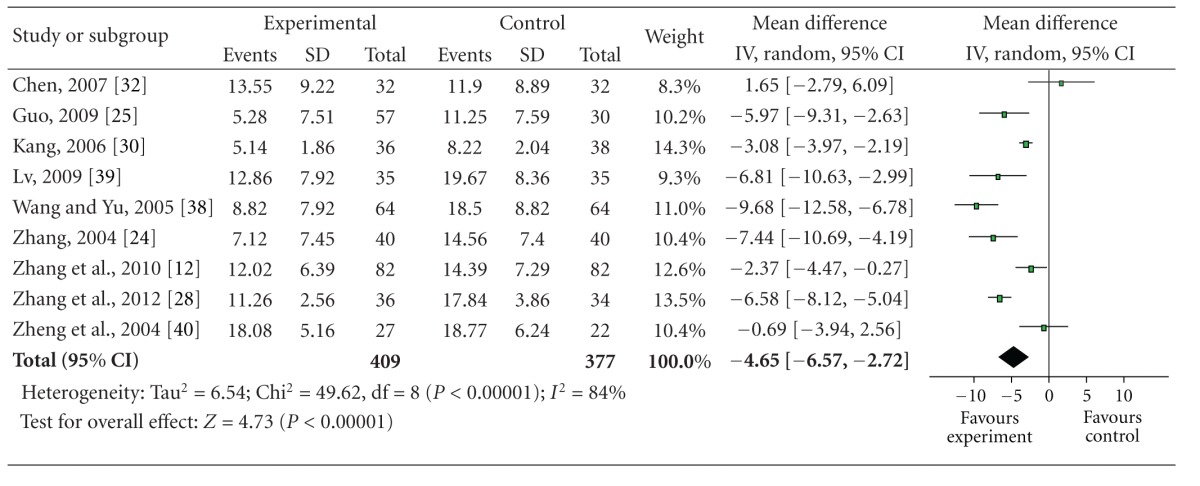
